# Quinine Pills

**Published:** 1901-03-30

**Authors:** 


### QUININE PILLS.

In the making of quinine pills the "British Pharmacopœia" orders that sulphate of quinine shall be triturated with tartaric acid in certain proportions and then added to a mixture of glycerine and tragacanth in powder, previously mixed. By this process a mass is made which will, it is hoped, at any time dissolve at the warmth of the body and in the presence of water. The glycerine, however, is essential, and if the dispenser skimps his glycerine he will as surely spoil pills as a cook would her pudding were she to spare her eggs; although for durability his pills will no doubt take front rank. Dr. Curnow[1] describes how a patient suffering from enteric fever, after twenty-two days of pyrexia of a very intermittent type, and obstinate constipation, was treated with 5 grains of quinine in the form of two pills three times a day. A week later, after a simple enema, the pills were found in the stool. They were seven in number, hard and apparently unacted on by the gastric or intestinal juices. It was subsequently ascertained that in making the pills water had been used instead of glycerine. The pills were not coated. Perhaps dispensers do not always realise how durable a compound may result from tragacanth and water, and it is not impossible that the apparent lack of efficiency which other drugs besides quinine sometimes display may be due to the strong mass in which they are administered.

[1] Lancet, March 23.

